# Metagenomic assemblies tend to break around antibiotic resistance genes

**DOI:** 10.1186/s12864-024-10876-0

**Published:** 2024-10-14

**Authors:** Anna Abramova, Antti Karkman, Johan Bengtsson-Palme

**Affiliations:** 1 Department of Infectious Diseases, Institute of Biomedicine, The Sahlgrenska Academy, University of Gothenburg, Guldhedsgatan 10A, Gothenburg, 413 46 Sweden; 2 Division of Systems and Synthetic Biology, Department of Life Sciences, SciLifeLab, Chalmers University of Technology, Gothenburg, 412 96 Sweden; 3 Centre for Antibiotic Resistance Research (CARe), Gothenburg, Sweden; 4 Department of Microbiology, University of Helsinki, Helsinki, Finland

**Keywords:** Metagenomic assembly, Antibiotic resistance genes, Genomic context

## Abstract

**Background:**

Assembly of metagenomic samples can provide essential information about the mobility potential and taxonomic origin of antibiotic resistance genes (ARGs) and inform interventions to prevent further spread of resistant bacteria. However, similar to other conserved regions, such as ribosomal RNA genes and mobile genetic elements, almost identical ARGs typically occur in multiple genomic contexts across different species, representing a considerable challenge for the assembly process. Usually, this results in many fragmented contigs of unclear origin, complicating the risk assessment of ARG detections. To systematically investigate the impact of this issue on detection, quantification and contextualization of ARGs, we evaluated the performance of different assembly approaches, including genomic-, metagenomic- and transcriptomic-specialized assemblers. We quantified recovery and accuracy rates of each tool for ARGs both from in silico spiked metagenomic samples as well as real samples sequenced using both long- and short-read sequencing technologies.

**Results:**

The results revealed that none of the investigated tools can accurately capture genomic contexts present in samples of high complexity. The transcriptomic assembler Trinity showed a better performance in terms of reconstructing longer and fewer contigs matching unique genomic contexts, which can be beneficial for deciphering the taxonomic origin of ARGs. The currently commonly used metagenomic assembly tools metaSPAdes and MEGAHIT were able to identify the ARG repertoire but failed to fully recover the diversity of genomic contexts present in a sample. On top of that, in a complex scenario MEGAHIT produced very short contigs, which can lead to considerable underestimation of the resistome in a given sample.

**Conclusions:**

Our study shows that metaSPAdes and Trinity would be the preferable tools in terms of accuracy to recover correct genomic contexts around ARGs in metagenomic samples characterized by uneven coverages. Overall, the inability of assemblers to reconstruct long ARG-containing contigs has impacts on ARG quantification, suggesting that directly mapping reads to an ARG database should be performed as a complementary strategy to get accurate ARG abundance and diversity measures.

**Supplementary Information:**

The online version contains supplementary material available at 10.1186/s12864-024-10876-0.

## Background

Antimicrobial resistance (AMR) is an increasing global health crisis causing hundreds of thousands of deaths each year worldwide [1]. To limit its spread, there is a need to identify and quantify resistance in both clinical and environmental settings. Metagenomic sequencing is a powerful tool allowing simultaneous identification and quantification of antibiotic resistance genes (ARGs) in each sample. Metagenomic analysis of sewage from different parts of the world has revealed that the same ARGs are found in different genomic backgrounds globally, proving the need to not only identify the composition of ARGs, but also in what genomic context they are present. The genomic background of an ARG determines co-resistance patterns and mobilization potential, both of which can affect the choice of intervention strategies locally and globally [2]. For this reason, metagenomic sequencing has been suggested as a possible means for surveillance of AMR not only in sewage [3, 4], but also in the environment in general [5]. Current high-throughput sequencing platforms produce hundreds of millions of reads that require assembly to be reconstructed into longer stretches called contigs, which can provide more contextual information. This step is typically demanding in terms of computational resources and time [6, 7]. On top of this, short read length, skewed species abundance distributions, high similarity between closely related ARG variants, and massive amounts of data make recovery of ARGs and the context around them challenging from metagenomic data [8, 9].

There are currently several tools available to assemble short-read sequencing data from metagenomic samples (see review by Ayling et al. [8]), most of which use variants of the de Bruijn graph approach to handle large amounts of data in an efficient way. This approach is based on reconstructing graphs to represent k-mers present in a set of reads, followed by traversing these graphs and identifying the most probable path representing a contig. Converting a graph path into a contig is not a trivial task. Metagenomic samples typically contain an unknown number of species with unknown abundance distributions. In the case of related species, sequences can carry similar sets of k-mers resulting in complex assembly graphs. This is further complicated by conserved repetitive regions, such as ribosomal RNA genes, mobile genetic elements (e.g. transposons and insertion sequences) and ARGs. Assembling conserved regions present in several different genomic contexts typically results in highly complex branched assembly graphs, which makes traversing the graphs extremely difficult. This is generally solved by splitting the graph into multiple short contigs [10]. For metagenomic analysis targeting ARGs, this means that sometimes all contextual information regarding the taxonomic origin or mobility of a gene will be lost, which can potentially lead to misinterpretation of the results.

There are several studies benchmarking metagenomic assembly tools, such as the “Critical Assessment of Metagenome Interpretation” (CAMI) challenge [6, 11]. The focus of these studies has largely been on the ability of assemblers to distinguish evolutionary related organisms in complex microbial samples. There are a few studies looking at resistome recovery from both real and simulated data using short-read, long-read and hybrid approaches. Yorki et al., [9] compared long- and short-read assemblers for recovering low-abundance species and resistance genes. Brown et al. [12] specifically investigated the ability of different assemblers to contextualize ARGs using co-occurrence of ARGs and mobile genetic elements (MGEs) on assembled contigs as a proxy. These studies provide a good overview of pros and cons of the different approaches in recovering ARGs from metagenomic data. However, a critical evaluation of currently available short-read assemblers for reconstructing the context around ARGs existing in multiple genomic contexts is currently lacking. More importantly, the impact of assembler choice on the biological interpretability has not been well explored.

ARGs constitute a type of genomic feature that is particularly likely to be fragmented in metagenomic assemblies, as they are often present in multiple contexts, can be surrounded by various forms of repeat regions, and can be present on plasmids with varying degrees of copy numbers. A specific investigation of how assemblers handle these genes is therefore warranted. Furthermore, the resulting assemblies are often used to perform ARG quantification by mapping reads back to the contigs to estimate gene abundances. It is not clear how the choice of assembler will affect this form of ARG quantification and, by consequence, the final biological interpretation of the results.

The main goal of this study was to systematically evaluate the capability of assembly tools to recover ARGs in the correct genomic context from metagenomic data. To have a controlled but still real-life relevant experimental set up, we first used a real data set from human stool samples and spiked it with simulated reads derived from plasmids containing ARGs. We then assembled the test datasets and evaluated performance of several tools (Velvet, SPAdes, metaSPAdes, MEGAHIT, Trinity, Ray) with respect to their accuracy of recovering the genomic contexts of ARGs using the original plasmids as reference. Furthermore, we did the same assessment but on a sample sequenced with both short- and long-read technologies, using the latter as a reference. The results provide important perspectives on the choice of assembly programs for recovering correct genomic contexts for ARGs from metagenomic samples. Furthermore, they call into question some of the practices currently used for quantification of ARGs based on metagenomic sequencing.

## Methods

### Evaluation using simulated reads

To obtain a controlled experimental setup, we randomly selected a metagenomic dataset generated from a real sample and spiked it with simulated reads derived from plasmids containing a known set of ARGs. The metagenomic dataset was downloaded from Sequence Read Archive (SRA) and corresponds to a human stool sample, representing a common sample type used for studies of AMR. This sample was sequenced by Illumina NextSeq550 with 150 bp reads, resulting in 4.1 Gb dataset (SRR9654970). To obtain a set of plasmids, we first chose a number of clinically-relevant and commonly observed ARGs from different ARG classes, including *sul2* (816 bp), *blaNDM-1* (813 bp), *blaTEM* (861 bp), *aph(3″)-Ib_3* (804 bp) and *tet(A)* (1200 bp). We downloaded protein sequences from the Comprehensive Antibiotic Resistance Database (CARD) database and used them as queries for NCBI BLAST database searches to retrieve complete plasmid sequences. Only hits with > 98% identity to the ARG query and corresponding to full-length plasmids were selected, five for each selected ARG (Table 1). We aimed to select plasmids of different sizes to reflect the diversity in natural samples.

**Table 1:** A list of plasmids chosen for the test and the ARGs they contain

ARG	Accession	Species	Strain	Plasmid	Length, bp
aph(3″)-Ib_3	CP039146.1	*Acinetobacter sp.*	10FS3-1	p10FS3-1–3	73,803
aph(3″)-Ib_3	CP058166.1	*Enterobacter hormaechei*	RHBSTW-00070	pRHBSTW-00070_3	9923
aph(3″)-Ib_3	CP026933.2	*Escherichia coli*	CFS3273	pCFS3273-1	268,665
aph(3″)-Ib_3	CP055808.1	*Escherichia fergusonii*	RHB03-C23	pRHB03-C23_3	35,135
aph(3″)-Ib_3	CP064948.1	*Pseudomonas fulva*	ZDHY414	pVIM-24-ZDHY414	589,460
blaNDM-1	AP023079.1	*Acinetobacter baumannii*	OCU_Ac16a	pOCU_Ac16a_2	41,087
blaNDM-1	CP055250.1	*Citrobacter freundii*	ZY198	pZY-NDM1	53,573
blaNDM-1	CP047406.1	*Escherichia coli*	MS6193	pMS6193A-NDM	142,890
blaNDM-1	CP050380.1	*Klebsiella pneumoniae*	51,015	p51015_NDM_1	353,810
blaNDM-1	CP040184.1	*Raoultella planticola*	Rp_CZ180511	pRpNDM-1	334,854
blaTEM-9	CP063225.1	*Enterobacter hormaechei subsp. Steigerwaltii*	BD-50-Eh	pBD-50-Eh_2	336,282
blaTEM-9	KR259131.1	*Escherichia coli*	EC3587	pEC3587	10,483
blaTEM-9	CP025144.1	*Klebsiella pneumoniae*	NR5632	NR5632_p1	204,123
blaTEM-9	GQ160960.2	*Serratia marcescens*		R934	13,775
blaTEM-9	CP024467.1	*Shigella dysenteriae*	BU53M1	unnamed1	54,993
sul2	CP059301.1	*Acinetobacter baumannii*	AC1633	pAC1633-1	174,292
sul2	CP055707.1	*Citrobacter freundii*	RHB16-C02	pRHB16-C02_6	6801
sul2	CP061493.1	*Enterobacter hormaechei subsp. Xiangfangensis*	GENC284	pGENC284	304,958
sul2	AP022550.1	*Escherichia coli*	THO-015	pTHO-015–1	88,121
sul2	MT415059.1	*Klebsiella pneumoniae*	NMI3243_13	pIncR_3243	69,560
tet(A)_1	CP047745.1	*Enterobacter hormaechei*	Eho-4	pEcl4-5	37,460
tet(A)_1	AP022535.1	*Escherichia coli*	THO-006	pTHO-006–2	101,966
tet(A)_1	CP064130.1	*Klebsiella pneumoniae*	M911-1	pM911-1.1	75,711
tet(A)_1	CP065164.1	*Klebsiella variicola*	KPN029	unnamed2	243,621
tet(A)_1	CP062224.1	*Salmonella enterica subsp. enterica serovar Goldcoast*	R18.1656	p270k	270,696

We used insilicoseq [13] to generate simulated reads from plasmids using the NovaSeq error model. Generally in metagenomic samples the coverage of a plasmid would vary depending on its number of copies and its size. To emulate this complexity, we provided an abundance.txt file containing proportions of reads weighed according to the size of each plasmid (smaller plasmids get more reads and larger less). Specifically, we used 1 × coverage of the largest plasmid among the selected plasmids (CP064948.1, 589,460 bp) as a baseline, which equals 1964 reads to cover the whole length one time. The number of simulated reads for each subsequent plasmid was estimated by multiplying 1964 reads to the length ratio. This resulted in a file containing 0.7 m simulated reads in total which was used to represent the baseline. To test how the total number of available reads from plasmids affected assembly process, we further generated several files with increasing amount of simulated plasmid reads by multiplying the number of reads corresponding to baseline by 2, 5 or 10 times, generating 1.4 m, 7.3 m and 14.7 m total plasmid reads per file. These files represent situations with low (0.7 m), medium to relatively high (1.4–7.4 m), and very high number of plasmid-derived reads (14.7 m), and will be denoted as “low”, “medium”, “high” and “very high” further in the text. 

As a result, we generated four files with different total amount of reads, as well as differential coverage, with the most abundant plasmid being 7000 × more prevalent than the least abundant. It is important to mention that despite being a simulated scenario, both the number of selected plasmids and their coverage is comparative to the complexity encountered in real samples. As an example, 26 known and 21 putative novel plasmids were recovered in an Indian lake metagenome [14]. In that study, plasmids of ~ 4500 bp, assembled from the metagenomic DNA sequenced by Illumina HiSeq2000, generated on average ~ 7000 reads per plasmid, which corresponds to > 460 × coverage (data not shown, generated based on Bengtsson-Palme et al., 2014).

**Fig. 1:**
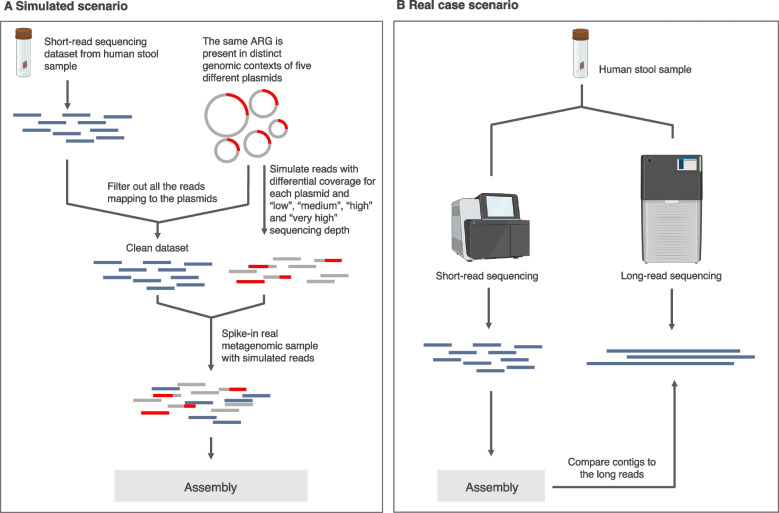
Workflow. **A** Simulated scenario constituting a real metagenomic dataset spiked with reads generated from a set of plasmids containing ARGs. **B** Real case scenario included long-reads which were used as a reference to quality check the contigs assembled from short read data generated from the same sample

In this study, we are only assessing the assembly quality of regions containing ARGs and not the quality of metagenomic assembly in general. Therefore, to ensure a controlled setup, reads from the human stool dataset were first mapped to the selected set of 25 plasmids and all matching reads were removed to create a clean test dataset (Fig. 1). The cleaned test dataset was then spiked with the simulated reads generated as described above. This resulted in a dataset with highly controlled plasmid and ARG content, while also maintaining the general complexity of metagenomic assembly, including e.g. memory limitations, which also influence the performance of the assembly algorithms.

For this evaluation, we decided to include several different tools: genomic assemblers SPAdes 3.13.0 [15], Velvet 1.2.10 [16] and Ray 2.3.1 [17]; metagenomic assemblers MEGAHIT v1.0.3 [18] and SPAdes 3.13.0 with the -meta option (also referred to as “metaSPAdes”), and transcriptomic assembler Trinity 2.1.1 [19]. We also tested TriMetAss as an alternative method to see if it can improve the outcome. TriMetAss is an extension to the Trinity software, which acts as a targeted assembler designed to assemble common and well-conserved genes occurring in multiple genomic contexts in metagenomic data [14].

We used METAQUAST 5.2.0 [20] to evaluate general assembly performance. The summary statistics and mapping rates are shown in Table S1 and Table S2 accordingly. Bandage 0.8.1 [21] was used to create assembly graphs visualization. The python package DNA Features Viewer 3.1.3 [22] was used to generate figures showing genomic background of ARGs.

### Evaluation using long-reads reference

For the second test with long-read data we used publicly available data from Jin et al. [23], corresponding to human fecal samples sequenced by both Illumina HiSeq X Ten platform with 150 bp-long reads (SRR10917786, 34.2 Gbp) and PacBio RS II (SRR10917776, 8.7 Gbp) (Pacific Biosciences of California, Inc., USA). We performed error-correction of the raw reads using Canu 2.2 [24].

To avoid creating erroneous contigs we decided to not assemble the long-read data but instead rely on the long error-corrected reads as they most likely represent the ground truth. First, we annotated the reads using BLASTN against the ResFinder database (2021) [25]. Only long reads containing full ARG sequences with at least 98% identity were retained. These reads were further clustered with cd-hit 4.8.1 [26] (95% identity) to create a less redundant reference for further comparison to the short-read contigs. We assembled the corresponding short reads with the same set of tools as mentioned in the previous section. The resulting contigs were mapped using BLASTN to the PacBio reads reference to assess accuracy.

To estimate how the differences in ability to assemble ARGs by different tools affect the downstream results, we performed ARG quantification. First, the Illumina reads were mapped to each assembly using bowtie2 2.3.5.1 [27] and the coverage was estimated for all full and truncated ARG hits (minimum 95% identity and 80% coverage) on the assembled contigs using the FARAO [28] *estimate_coverage* function with -c 0 flag estimating coverage per all bases across the feature. All plots were created in R using ggplot2 [29]. Bray–Curtis dissimilarity calculation and analysis of variance were done using vegan (2.6–4) package.

### Assessment

To assess the performance of the tools, we identified contigs produced from the short-reads containing ARG sequences and estimated the number of fully assembled (100% coverage, 98% identity), truncated (minimum 60 bp length, 98% identity and no flanking regions on either of the sides or both sides) and misassembled/partial ARGs (minimum 60 bp, 98% identity, and embedded in incorrect flanking sequences). The 60 bp threshold was chosen because no significant similarity was found between the chosen ARGs at this length, and 60 bp should be sufficient to unambiguously detect a resistance gene in the simulated scenario. Furthermore, we investigated the genomic contexts of the fully assembled ARGs and whether they fully matched the original context by inspecting alignments to the original plasmids.

## Results

### Complex short-read data yield incomplete assembly of ARGs and their contexts

General assembly performance statistics (Additional file 1: Table S1) showed that overall genomic assemblers, in particular Velvet and Ray, performed worse in comparison to the metagenomic assemblers. Among metagenomic assemblers, MEGAHIT generated fewer and shorter contigs in comparison to metaSPAdes. A comparison of the length distributions between contigs generated from reads in the simulated data and the rest of the assembly highlights that, in general, the assembly tools perform with a different degree of success assembling complex regions independent of the general assembly performance (Additional file 1: Figure S1).

**Fig. 2:**
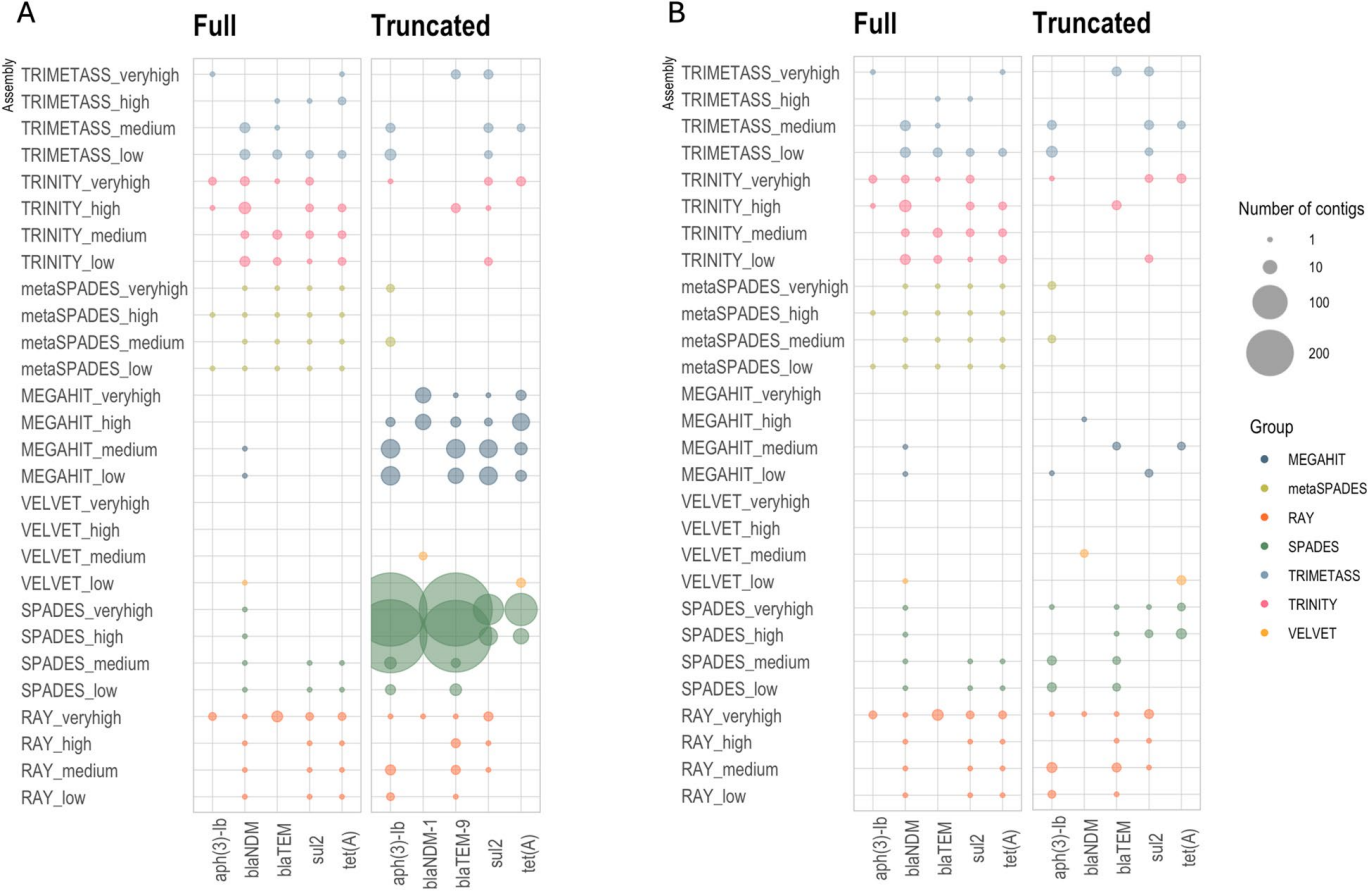
ARGs recovery by each tool. **A** Presence/absence of ARGs on the contigs assembled by different tools. **B** Presence/absence of ARGs after filtering using a length cut-off of 300 bp was applied to the results. “Full” denotes contigs containing full length and correctly assembled ARGs while “Truncated” comprises contigs containing partial ARG sequence (minimum 60 bp and 98% identity and no flanking regions on either of the sides or both sides)

To investigate which assemblers managed to reconstruct ARGs from short-read data, we first looked at the recovery of both full length and truncated ARG sequences (Fig. 2). For the simulated test data, the knowledge of exactly which ARGs were present on the original plasmids allowed us to precisely determine how many of those were correctly recovered by each assembler (Fig. 2A). For this analysis, we were interested in whether an ARG was recovered, even if it was assembled in the wrong context, because for some applications it is sufficient to just obtain the individual gene sequence correctly, regardless of context.

**Fig. 3:**
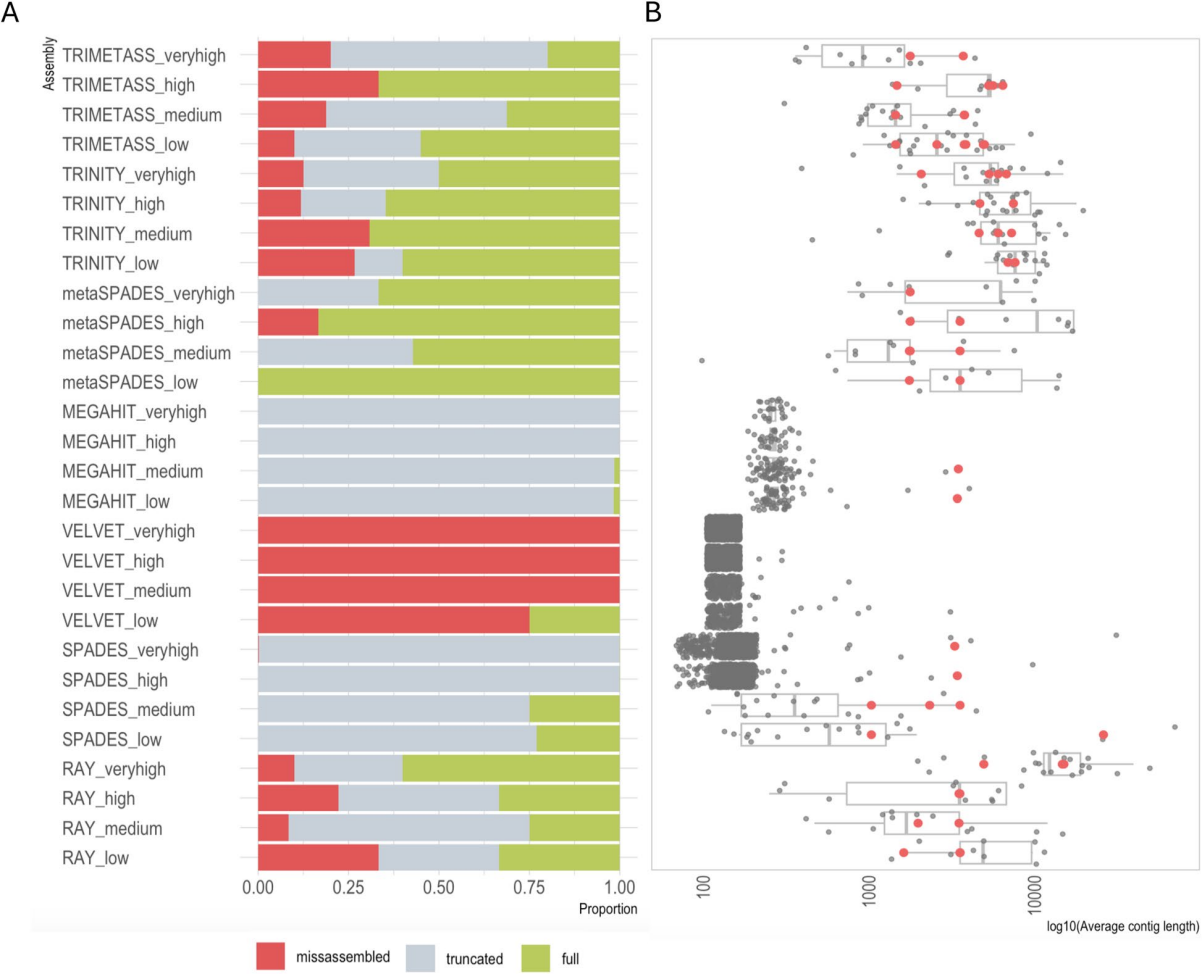
Assembler performance at different coverages. **A** Proportion of full, truncated and misassembled/partial ARG sequences. Note that the retrieved ARGs are not necessarily associated with the correct context.). **B** Length distribution of contigs with ARG hits. Contigs with correct genomic context, only containing full ARGs, are marked with red dots

The results showed that MEGAHIT, metaSPAdes and Trinity managed to capture almost all ARGs at all coverages. However, the MEGAHIT contigs containing ARGs were on average only 284 bp long, resulting in predominantly truncated ARG sequences (Figs. 2 and 3). In contrast, Trinity performed consistently better at all coverages, with more than 50% of contigs containing the full length ARG sequences (Fig. 3). While Trinity could assemble longer contigs compared to metaSPAdes, the number of misassemblies was higher. In all cases, except the “high” dataset, metaSPAdes did not produce misassembled ARGs. Among the genome assemblers, Ray had the best performance in terms of reconstructing full ARGs, while Velvet reconstructed only one full ARG out of 3724 assembled ARG-containing contigs, with the rest containing misassembled ARGs. SPAdes struggled to assemble ARGs at higher coverages, producing truncated contigs.

Furthermore, we investigated the number and length of correctly assembled contexts for the different assemblers. Figure 3B shows that Trinity performed better in comparison to the other tools, reconstructing on average longer correct contigs with quite consistent performance across the coverages. Notably, the performance of Ray was rather similar to that of metaSPAdes. In some cases, Ray produced even longer correct contigs despite being a genomic assembler not optimized for complex metagenomic samples. In contrast, MEGAHIT produced only two correct contigs at lower coverages. We also used SPAdes contigs as seeds to extend them using TriMetAss. The results revealed that in general TriMetAss output a few more correctly assembled contigs containing full ARGs. Importantly, these contigs were on average 2000 bp longer than initial SPAdes contigs. As a drawback, TriMetAss also produced more misassembled contigs in comparison to SPAdes output (Fig. 3A).

**Fig. 4:**
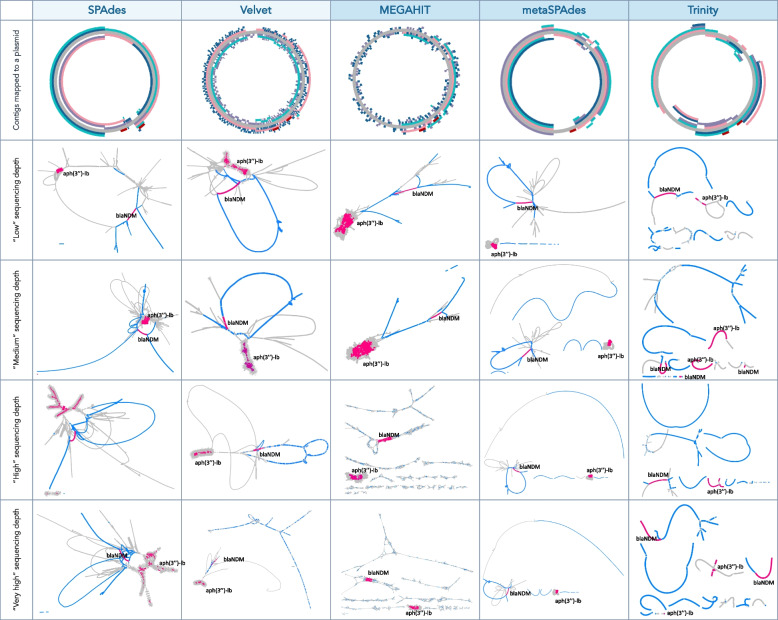
Visual representation of assembly results. The example used is one of the plasmids AP023079, containing two ARGs *blaNDM* and *aph(3’’)-Ib*. A visual representation of the plasmid was done using FARAO with light gray representing the backbone plasmid and the other colors representing correctly assembled contigs from samples with different total number of reads (“low” in pink, “medium” in teal, “high” in purple and “very high” in blue) and ARGs are in red. A visual representation of the corresponding assembly graphs was done using Bandage. The figures represent only part of the whole assembly graph corresponding to the AP023079 plasmid sequence, where blue lines correspond to BLAST hits of the assembled contigs to the plasmid and pink lines to the ARG regions

**Fig. 5:**
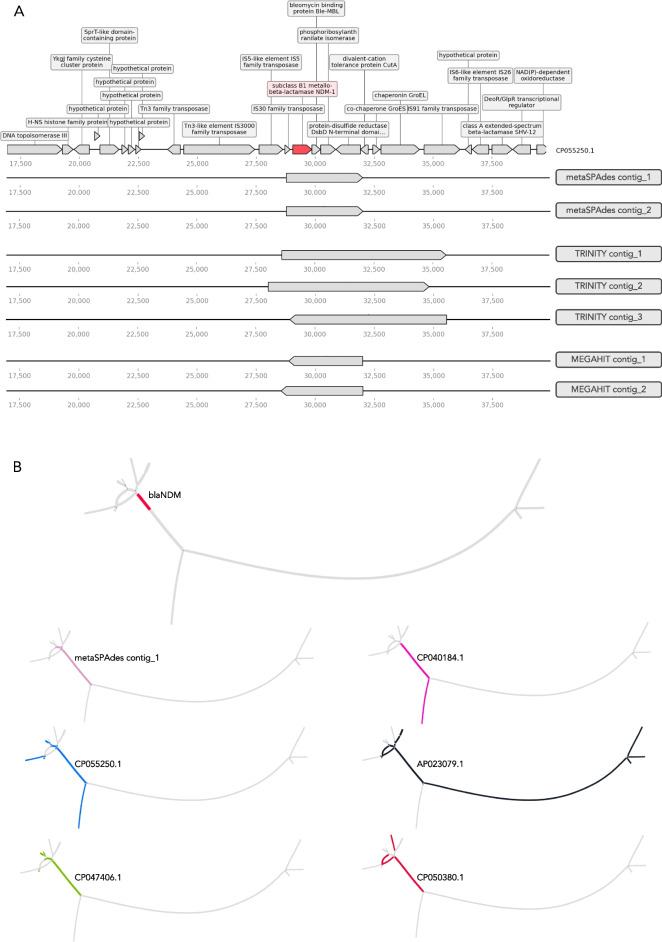
Assembly of *blaNDM-1* gene. **A** Alignment of the contigs containing *blaNDM-1* gene to a reference plasmid. The top figure depicts a part of the reference plasmid CP055250.1 with 10 kb upstream and downstream of the *blaNDM-1* gene (in red). Coding sequences of neighboring genes are shown as grey arrows. Note that the contigs start and/or end within genes encoding transposases or insertion sequences (ISs). **B** The top panel represents a part of the assembly graph for the “medium” metaSPAdes assembly containing *blaNDM-1* gene (in red). Each subsequent graph shows the mapping of the output contig as well as paths corresponding to the original plasmid sequences

What is obvious and rather surprising is that from a total number of 25 different genomic contexts for the 5 different resistance genes present in the sample on average only three original contexts (12%) were correctly captured by any of the assembler. These contexts corresponded to plasmids of different sizes suggesting that the total length of plasmids did not determine assembly success, but rather features surrounding the particular genes (Figure S2). All assemblers reconstructed large contigs spanning in some cases half of the plasmid sequence (as shown on the example of AP023079 plasmid; Fig. 4), but these assembled contigs broke exactly at the beginning of MGEs and/or ARG sequences (Fig. 5A). The complexity of assembly graphs also increased with more coverage, but for some tools, such as SPAdes and metaSPAdes, additional coverage helped to resolve ambiguous branching and reconstruct longer contigs, while for MEGAHIT increased coverage resulted in profound fragmentation. The assembly graphs also made it clear that Trinity, as a transcriptomic assembler, utilizes a different approach in comparison to the other tools, resulting in very characteristic assembly graph patterns.

### Long-read data confirmed results of simulated metagenomes

To validate the findings from the simulated metagenome data, we performed a second test with a real dataset. For this, we used PacBio reads containing full ARGs as a reference for the contigs assembled from a corresponding short-read data set derived from the same samples (see Methods for details). In this dataset, we annotated ARGs on the PacBio reads, which resulted in 18 unique ARGs (98% identity and 100% coverage) found on 125 PacBio reads (32 reads carried more than one ARG). This set of PacBio reads was used as a reference to evaluate contigs containing ARGs assembled from short-read data. Short-read and long-read datasets used for this test are different in terms of the sequencing depth, which might affect the detection of ARGs. Therefore, for the genomic context comparisons, we only focused on the comparison of those contigs that aligned to the reference read present in the PacBio long reads data. That is, if there was a contig assembled from short-reads that did not align to the PacBio reference it was not taken into account, to avoid misinterpretation due to difference in sequencing depth.

**Fig. 6:**
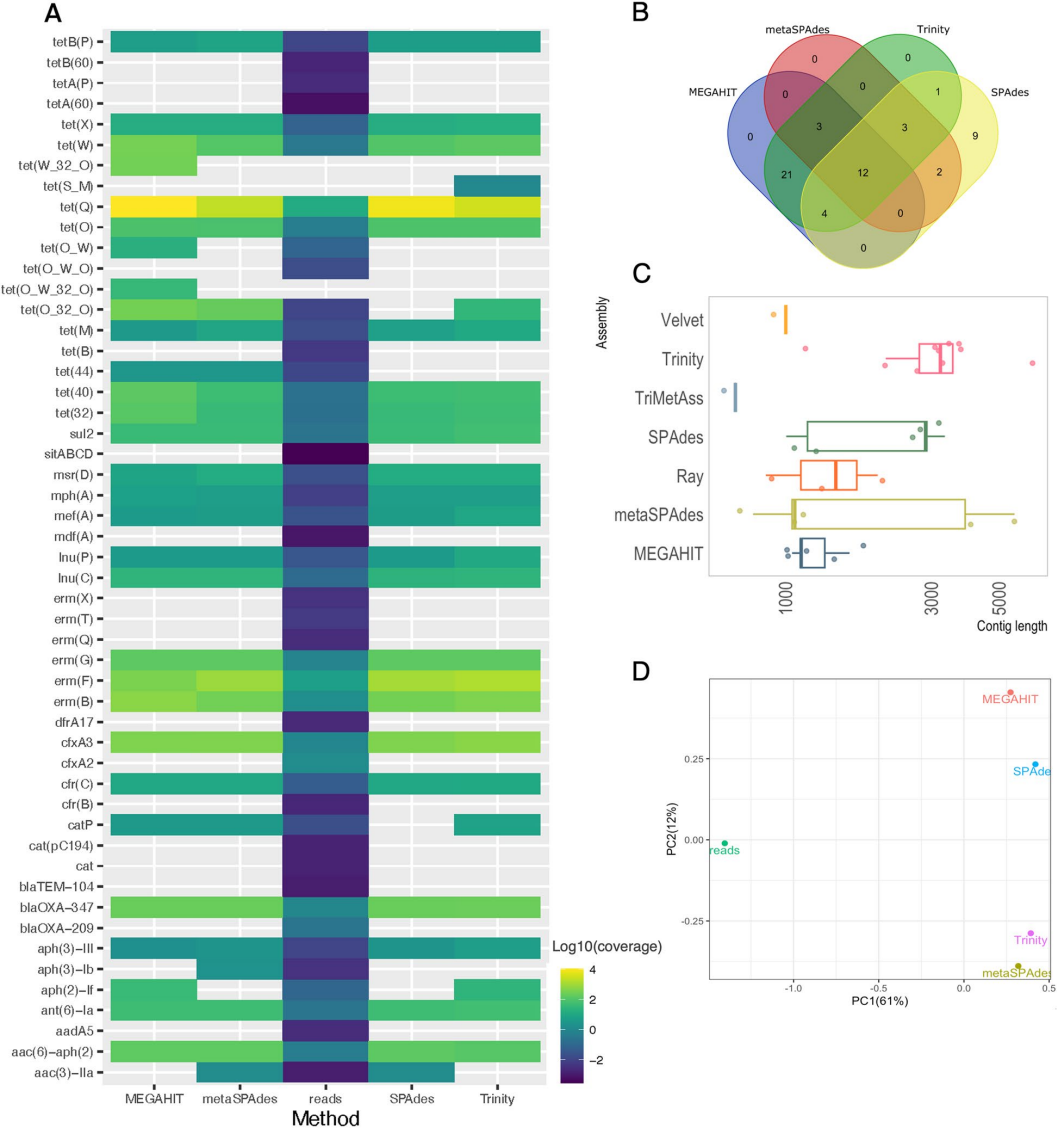
Results from comparison between short and long read data. **A** ARG quantification by using either assembled contigs or direct mapping of short reads to the ResFinder database, represented as log10(per base coverage). **B** Number of unique ARGs identified on assembled contigs (Ray, Velvet and TriMetAss are not shown). **C** Length distribution of contigs assembled from short reads matching the PacBio reference reads, with dots representing individual contigs. **D** PCoA based on Bray–Curtis dissimilarity between different quantification methods

After assembling the short-reads data, we compared these contigs to the PacBio reference reads to assess the correctness of genomic context. In this comparison, Trinity had the highest number of correct contigs matching the reference PacBio reads, and these contigs were on average longer than the ones reconstructed by the other tools (Fig. 6C and Table S3). In contrast, MEGAHIT, metaSPAdes and SPAdes assembled half as many contigs with on average shorter length than Trinity. To check if another approach using TriMetAss could improve the results, we used SPAdes contigs containing full and truncated ARGs as a seed for iterative re-assembling. However, the results revealed that this approach did not considerably improve the length of the contigs containing ARGs.

Altogether, 55 unique ARGs were identified from the assembled contigs by all the short-read tools, with only 12 ARGs common between all of them (Fig. 6B). Interestingly, Trinity and MEGAHIT had the most number of common ARGs. Importantly, annotation of short reads alone resulted in 85 matches (80% coverage and 98% identity).

### Abundances of ARGs depends on the approach used for quantification

**Fig. 7:**
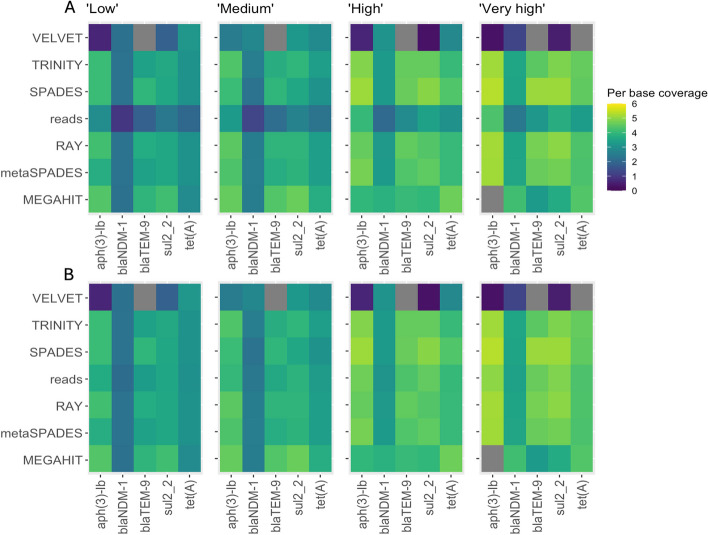
ARG quantification using either assembled contigs as a reference or by directly mapping short reads to the ResFinder database**.** Per base total coverage calculated using FARAO from aligning reads to the contigs, and using direct ARG quantification by mapping reads to the ResFinder database (**A**) and ResFinder clustered to 90% identity (**B**)

To investigate what consequences assembly fragmentation has on the ARG quantification, we mapped reads back to the corresponding assemblies to estimate gene abundances. In parallel, we quantified ARGs by mapping reads directly to the ResFinder database (Fig. 7A) and to the same database but clustered by 90% identity to reduce variant redundancy (Fig. 7B). This provides a direct comparison between the two prevailing approaches to quantify ARGs in metagenomic data [10].

In the simulated scenario, Velvet, and to some extent MEGAHIT, showed a read mapping pattern that was inconsistent with the other assemblers, largely due to their inability to produce contigs containing complete ARGs. This effect was larger in the ‘high’ and ‘very high’ total plasmid read cases. Furthermore, comparing mapping results between ResFinder before and after clustering revealed that for the investigated ARGs the abundance estimates are lower for the non-clustered database.

For the real dataset case, abundance calculation revealed that for some ARGs, results are very similar between approaches, such as for several tetracycline genes (e.g., *tet(Q)* and *tet(W)*), as well as the erythromycin resistance gene *erm(B)* and aminoglycoside resistance gene *aph(3’)-III* (Fig. 6A). However, several ARGs were detected only by mapping reads directly to the ResFinder database and were missing completely from the assembly-based approach (e.g., *tet(B)*, *blaTEM-104* and *erm(T)*). At the same time, the abundances for many ARGs were lower when quantified directly against the ResFinder database than they were when quantified by mapping to contigs. Principal coordinate analysis using Bray–Curtis dissimilarity on abundances estimated by different methods revealed that results from different assembly methods were not dramatically different, but direct reads quantification was substantially different from the methods mapping reads back to the assembly to assess ARG abundance (Fig. 6D). To a large extent, this is due to that direct read mapping is able to detect more different types of ARGs that the assembly-based methods (Fig. 6A).

Taken together, this indicates that direct read mapping might better capture the diversity of ARGs in a metagenome by detecting rare genes not present on the assembled contigs, but may at the same time underestimate the abundances of more common ARGs.

## Discussion

### The repertoire of ARGs recovered from the same dataset differs depending on the tool used

It is commonly assumed that metagenomic high-throughput sequencing allows an *unbiased* cataloging of ARGs at the whole microbiome scale [30, 31] in comparison to qPCR and culture-based methods. However, it is rarely considered that downstream data processing can have major impacts on the results reported.

There have been several initiatives to benchmark assembly software for metagenomics. Wang et al. [11] performed evaluation of metagenomic assemblers using real metagenomic datasets spiked with reads from known genomes focusing on completeness and accuracy of reconstructed genomes. A few studies have looked into the benefits of using long-reads for improving metagenomic assemblies [32–34]. Within the framework of the CAMI challenge, Sczyrba et al. [35] and Meyer et al. [6] evaluated assembly performance, but largely for the purpose of taxonomic profiling. However, only a few studies have investigated the implications of assembler choice for the inference of gene contexts and gene abundances from metagenomic assemblies. Brown et al. [12] used resistome risk score based on co-occurrences of ARGs, MGEs and pathogen gene markers on the same contig to evaluate convergence of biological output produced by different assemblers. Another paper by Galata et al. [36] showed that the choice of assembly software as well as sample complexity have considerable impact on prediction of genes and proteins. A recent study by Yorki et al. [9] focused on assessment of short-, long- and hybrid-approaches to recover the genome of clinically relevant low-abundant *E. coli* and their ARG content from metagenomic samples. Unfortunately, the results of these studies are often contradictory, most probably due to the different approaches and features of the underlying test data. Most importantly, despite being mentioned by several studies, the impact of assembler choice on the biological interpretability has not been well explored.

In the current study, we used both simulated and real metagenomics data to assess the impact of assembler choice on the identification and quantification of ARGs, as well as the ability to correctly reconstruct the genomic contexts surrounding these ARGs. Metagenomic assemblers are optimized to deal with sequence data from samples containing multiple species in different abundances, and therefore their performance was of primary interest. In the simulated scenario, metaSPAdes considerably outperformed MEGAHIT in terms of number of contigs containing full ARG sequences, with MEGAHIT predominantly produced short contigs (on average 284 bp long) with truncated ARGs. This could have severe consequences on the results since many metagenomic studies utilizing the assembly approach employ a filtering step to remove short, potentially erroneous, contigs containing little useful information. The filtering cut-off can vary from 300 bp up to 2 kb [37–40]. Even if we were to apply the most allowing cut-off of 300 bp to the MEGAHIT results, 99% of contigs containing ARGs would be filtered out, resulting in a considerable underestimation of the resistome in the sample. This suggests that the choice of assembler as well as pre-processing and post-processing steps can considerably affect the outcomes and interpretations of a study.

This problem became even more profound when we performed ARG identification in a real dataset. For a real dataset, there is no way to know the true complete repertoire of ARGs. Therefore, we used PacBio reads containing full ARGs as a reference for the contigs assembled from corresponding short-read data. Altogether, 55 unique ARGs were identified from all the assembled contigs across all the short-read tools and the PacBio reads (Fig. 6B). The number of unique ARGs captured by the different assemblers varied greatly, from 44 identified by Trinity to 20 captured by metaSPAdes. Similarly to the assemblies from the simulated data, 52% of the MEGAHIT contigs containing ARGs would have been filtered out using a 300 bp length cut-off, showing that this undesired effect is not simply a matter of our methodological choices for simulating data. Trinity, SPAdes and metaSPAdes recovered several additional ARGs not present on the PacBio reads. In this case, the short-read dataset was sequenced three times deeper than the PacBio dataset, suggesting that the long-read dataset did not have sufficient depth to pick up all the ARGs. That said, the two approaches would probably perform similarly well at comparable sequencing depth, but the costs of long read sequencing would – at present – be considerably higher.

Worryingly, only six ARGs were commonly identified by all tools and on PacBio reads, including two aminoglycoside, two tetracycline and two erythromycin resistance genes. Consequently, some genes were missing from the output by all short-read assemblers tested, including the clinically relevant beta-lactamase gene *blaOXA-209*, which was identified on PacBio reads and therefore most certainly present in the sample. The most probable explanation for this is that those were relatively rare genes that did not get sufficient coverage to be assembled by the short-read sequencing effort and therefore are missing from the resulting assembly. An alternative approach to ARG quantification in metagenomes, circumventing assembly, is identification of ARGs by mapping the reads directly to one of the available ARG databases [10]. We annotated reads by mapping reads to the ResFinder database, which resulted in identification of 85 unique ARGs. Perhaps not surprisingly, this number by far surpassed the total number of ARGs identified by mapping reads to the assembled contigs. Reads are typically much shorter than contigs and might map spuriously to several different targets causing false positives. At the same time, this approach does not require coverage of the entire ARG to detect it, which may be crucial for the detection of rare ARGs. As many clinically relevant ARGs to last resort antibiotics are typically rare in most microbiomes, the increase of detection ability is highly important for e.g. monitoring of high-risk ARGs [5, 41]. This finding also highlights the importance of not basing gene catalogs only on assemblies from the metagenomes under study, but also including relevant gene or genome repositories into the catalogs used for annotation and read mapping[42].

Depending on which tool and cut-off are used for the data analysis, the end results can be drastically different. It is important to mention, though, that the number of correctly assembled full-length ARGs on its own is not always a good measure of assembler performance. For example, the rest of the output contigs may contain misassembled sequences, which is an undesired outcome. In most real-world scenarios, it would not be possible to determine which contigs were correctly and incorrectly assembled, underscoring the importance of assembly tools achieving a good ability to stitch reads together while still maintaining strict precision in terms of obtaining the correct assembled contexts using default settings.

### Correctly assembled short contigs often lack context around ARGs

Obtaining a correctly assembled full or even truncated ARG might be enough for certain applications, for example when estimating the ARG diversity in a sample. However, for the purpose of host taxonomic inference or mobility assessment of a given ARG, it is necessary to correctly identify the genomic context around it. After assembling short-reads data we compared the resulting contigs to the original plasmid sequences for a simulated data set or to PacBio reference reads for the real data scenario, allowing us to assess the correctness of the assembled genomic contexts. In this comparison, Trinity had the highest number of correct contigs matching the reference in both cases, and the Trinity contigs were on average longer than the ones reconstructed by the other tools (Fig. 3B and Table S3). Trinity is a transcriptome assembler, specifically designed to assemble transcript variants resulting from alternative splicing or gene duplication [43]. In contrast with most short-read assemblers that typically collapse the variant features into consensus sequences, Trinity aims to capture the diversity of splice isoforms by first assembling disjoint ‘transcription loci’ that are further converted into de Brujin graphs and pruned based on read support. The graph representation in Fig. 4 shows that Trinity contigs are represented by nodes of more even coverage and less complexity in comparison to graphs resulting from metaSPAdes and MEGAHIT assemblies. This transcriptomic assembly approach is similar to the rapidly emerging localized assembly graph approaches developed to leverage variants present in complex microbial communities. This can be achieved by extracting parts of the sequence graph generated during metagenomic assembly surrounding a region of interest and using coverage statistics to validate the genomic contexts [44–46].

In contrast to Trinity, the short-read metagenomic assemblers MEGAHIT and metaSPAdes recovered on average fewer and shorter contigs with correct genomic contexts. The original MEGAHIT publication [47] showed that its performance becomes better with increased coverage (from 10 × to 100 ×) in terms of N50 value, largest alignment length and number of misassemblies. However, we observed that in our simulated data scenario, MEGAHIT performed best at lower coverages (Figs. 2 and 3), showing extensive fragmentation at the higher coverages as revealed by the highly branching graph (Fig. 4). In the real data scenario MEGAHIT and metaSPAdes showed very similar performance in terms of number of correct contigs and their length. This is somewhat reflective of our simulated approach representing a very complex, but yet realistic, case in terms of the number of different resistance plasmids present in the simulated data. That said, due to the rather short length of the MEGAHIT and metaSPAdes contigs they match to several different PacBio reads (different genomic contexts) implying that the length of most of them is not sufficient to unambiguously decipher the taxonomic origin of the ARGs they carry. Considering that the average ARGs is longer than 500 bp, these contigs most probably also lack any information about co-located ARGs or MGEs, at least with any degree of certainty. This becomes even more apparent when looking at the part of a graph from “medium” metaSPAdes assembly in the simulated scenario (Fig. 5B). This assembly resulted in only one contig containing the full *blaNDM-1* gene and representing a common region between the five original plasmids. However, from the assembly graphs it is clear that there are several longer potential graph paths corresponding to the original plasmids. Therefore, simply relying on the metagenomic assemblers for the purpose of capturing genomic contexts, especially in complex metagenomic samples, might lead to considerable underestimation of the diversity present in a sample. Using graph-based approaches would likely be more suitable for that task, and recent developments in this direction, including GraphAMR [48] and MetaCortex [49], have shown some promising results and may eventually be interesting to evaluate in the same way as the assemblers tested in this study.

### Quantification of ARGs is dependent on correct assemblies

As has been discussed above, the repertoire of ARGs detected in the assemblies varied greatly between different assembly tools in both scenarios. To investigate what consequences this has on the ARG quantification, we mapped reads back to the corresponding assemblies to estimate gene abundances. In parallel, we quantified ARGs by mapping reads directly to the ResFinder database. For the simulated scenario, we have knowledge about exactly which ARG sequences should be present in the dataset and we can therefore can directly compare abundances for these genes estimated by two different approaches. It was surprising to observe that quantification by mapping reads back to the ResFinder database revealed in general lower abundance levels than when calculating abundance by mapping to the assembled contigs (Fig. 7A). The ResFinder database, as well as the other AMR gene catalogs, contains two hierarchical levels of nomenclature: gene family such as *blaNDM* or *tet(M)* and their associated allelic variants (e.g. *blaNDM-1*, *tet(M)-6*). We hypothesized that the observed results is a consequence of variant ”spill-over” effect when the lengths of the reads were insufficient to differentiate between the variants. A possible solution often used to reduce the impact of this variant”spill-over” effects is to cluster all the similar variants and retain only a single representative sequence. We did this in an additional test using the ResFinder database clustered to 90% identity (Fig. 7B). This approach allowed us to estimate the abundance for a family of closely related ARG variants instead of a particular variant. Despite the somewhat lower resolution with regards to specific ARG variants, using a clustered database yielded abundance estimates for all the spiked-in genes comparable to those estimated based on the mapping to assembled contigs.

In the real-case scenario, the results revealed that several ARGs were quantified only by mapping short reads directly to the ResFinder database and were missing completely from the assembly-based approach (Fig. 6A). Some of those are clinically relevant genes such as *tet(A)*, *tet(B)*, *blaTEM* and *erm(T)*, and therefore it is crucial to understand if their presence/absence is an artifact. In the case of *blaTEM*, it was only identified by mapping the short reads to the ResFinder database, and it was missing from assembled contigs. A closer inspection of the original Illumina dataset showed that it is a low-abundant gene that did not have enough coverage to be assembled by short-read assemblers.

All together, these results show that none of the approaches give a comprehensive picture of ARG diversity and abundance. Assembled contigs provide a good resolution in terms of identification and quantification of specific ARG variants as well as their genomic context, but at the same time this approach misses rare ARGs due to insufficient coverage. In contrast, direct mapping of short reads spuriously aligns them to different ARG variants, leading to an overestimation of the resistome diversity in a sample (and potentially also underestimation of gene abundances). At the same time, direct read mapping has the ability to identify rare genes not present on the assembled contigs due to lack of complete coverage. In addition, there are several studies suggesting the importance of knowing the genomic context of ARG variants for determining their transmission potential, co-resistance patterns and how well they would respond to different interventions [2, 50]. Therefore, using read-based quantification alone to determine ARG abundance in a sample can result in a misleading interpretation regarding which particular ARG variants are present and abundant in a sample. This highlights the importance of using a combination of approaches to obtain an unbiased picture of ARG diversity and abundance in a metagenomic sample and to exercise caution when interpreting individual ARG results from metagenomic data.

### Certain ARG contexts are particularly hard to assemble correctly

Interestingly, not all of the ARGs were equally easy to assemble (Fig. 2), with *aph(3’’)-lb* being the most difficult gene to fully assemble among the ones spiked-in. This is a good example of what happens during assembly of regions which are present in multiple genomic contexts with differential coverage in the same sample. On the original plasmids, the aminoglycoside resistance gene *aph(3’’)-lb* was flanked by insertion sequences, several other ARGs and recombinases (Additional file 1: Figure S4), all contributing to making it difficult to assemble the region around this gene correctly. In contrast, there are comparatively fewer insertion sequences in the vicinity of *blaNDM-1* (Fig. 5A and Additional file, Figure S5). The majority of the assembly tools produced contigs containing the full *blaNDM-1* gene but broke exactly at the insertion sequences (Fig. 5A).

A factor further complicating metagenomic assembly is that microbiomes are typically characterized by different abundance levels of various species. As a result, DNA sequencing yields a highly non-uniform distribution of read coverages across different genomes making it even more difficult to resolve assembly graphs. Not surprisingly, the assembly graphs showed that this problem becomes more pronounced with increasing coverage (Fig. 4, the brush-like structures representing *aph(3’’)-lb*). In a nutshell, this problem is analogous to the recovery of other conserved repetitive regions such as 16S rRNA genes from metagenomic samples. The 16S rRNA is a gene consisting of a patchwork of hypervariable and universally conserved regions, resulting in highly complex branched assembly structures [7]. In addition, read coverages for most species are much lower than in a typical cultivated single-species sample. All together, these features of metagenomic data cause standard genome assembly procedures to produce fragmented and error-prone assemblies, as can be seen in the examples of Velvet, Ray and SPAdes.

## Conclusions

Overall, there is a need for better assembly software to deal with ARGs in multiple contexts, as the results of this study show that none of the current tools can deal with samples of high complexity. Currently available metagenomics assembly tools metaSPAdes and MEGAHIT are able to identify a variety of ARGs but fail to fully recover the diversity of genomic contexts present in a sample. The transcriptomic assembler Trinity, despite being designed for a different purpose, is an interesting alternative as it showed better performance in reconstructing longer and fewer contigs matching unique genomic contexts, which can be beneficial for deciphering the taxonomic origin of ARGs. Therefore, for situations where a complex metagenome can be expected, we would recommend using Trinity. However, often the available computational resources will not allow this, as Trinity is a computationally very demanding software. As a second option, we suggest metaSPAdes, which also requires a lot of resources, and therefore is feasible only for smaller datasets, unless substantial computational resources are available. MEGAHIT showed quite poor performance in a complex case scenario, producing very short contigs, but its performance was comparable to that of metaSPAdes in the real data scenario. We would suggest MEGAHIT as an option for low complexity samples as it is also much more CPU- and memory-friendly than all other approaches. In addition, MEGAHIT might sometimes be the only feasible option for producing assemblies from very large datasets. This is not an ideal situation from a point of view of assigning contexts to ARGs. Currently emerging graph-based approaches show promising indications that they might be more suitable for that task.

Finally, we have made one very important observation: our results show that using a length filtering threshold for the assembled contigs can contribute to a dramatic loss of ARG-containing contigs. This is due to that ARGs seem to be over-represented among challenging genomic contexts for assembly, and for that reason these regions are particularly prone not to be properly assembled, resulting in short and fragmented contigs. This can lead to drastic underestimation of the resistome diversity and abundance in a sample. We suggest, therefore, to annotate ARGs on contigs *before* filtering on the length, to have an idea of what is being filtered out. When it comes to ARG abundance quantification, direct mapping of reads to a database rather than an assembly results in better detection ability, but risks increasing false positive detections. An alternative approach is to cluster the reference database to reduce the number of ARG variants. This will lead to a lower resolution at the ARG variant level but will on the other hand reduce the risks for biasing the picture of ARG prevalence. Another way could be to assign a threshold for the minimal number of reads mapped, distributed across a reference ARG, to make sure that there is enough support for that gene being present in a sample. Ultimately, the most pragmatic solution may be to both quantify ARGs by mapping reads directly to a database and by mapping reads to assembled contigs to determine coverage, as the approaches are somewhat complementary.

In conclusion, as researchers we should not blindly trust the output of our bioinformatics tools. If tools corroborate each other, one can put more trust into their output. If not, one should exercise caution when interpreting data, especially on genetic contexts in potentially complicated regions. Long read sequencing may eventually solve these problems, but we are not there yet, partially because of the excessive costs of deep long-read sequencing. In the meantime, new more accurate methods are needed to resolve the contexts around ARGs to determine where they belong taxonomically and their potential for mobility.

## Supplementary Information

Additional file 1

## Data Availability

The datasets analysed during the current study are available in the SRA database: https://www.ncbi.nlm.nih.gov/sra/?term=SRR10917786, https://www.ncbi.nlm.nih.gov/sra/?term=SRR9654970.

## Abbreviations

ARGs: Antibiotic resistance genes
AMR: Antimicrobial resistance
RNA: Ribonucleic acid
CAMI: Critical Assessment of Metagenome Interpretation
MGEs: Mobile genetic elements
SRA: Sequence Read Archive
Bp: Base pairs
CARD: Comprehensive Antibiotic Resistance Database
NCBI: National Center for Biotechnology Information
qPCR: Quantitative Polymerase Chain Reaction
