# Supplementary material for: Metagenomic assemblies tend to break around antibiotic resistance genes
Source: BMC Genomics. 2024 Oct 14;25:959. doi: 10.1186/s12864-024-10876-0 (PMC11479545; doi:10.1186/s12864-024-10876-0)
Supplement: Supplementary file 1 — Additional file 1 [file 12864_2024_10876_MOESM1_ESM.pdf]

Additional File 1

Table S1. METAQUAST reports for the "Original dataset" assemblies referring to a human stool sample (SRR9654970), "Simulated scenario" assemblies with different number of spiked-in plasmid-derived reads (LOW, MEDIUM, HIGH and VERY HIGH) and "Real data scenario" assemblies derived from SRR10917786 dataset (see Materials and Methods for more details).

Original dataset	Assembly statistics					
	Velvet	Ray	SPAdes	MEGAHIT	metaSPAdes	Trinity
# contigs	31,642	16,949	36,612	51,042	40,188	58,181
Largest contig	25,401	375,467	201,769	50,052	688,922	202,636
Total length	35,218,493	36,878,634	73,894,390	65,767,600	70,480,576	91,772,192
N50	1,223	7,323	4,637	1,574	3,148	2,330

Simulated scenario						
LOW	Velvet	Ray	SPAdes	MEGAHIT	metaSPAdes	Trinity
# contigs	32,218	17,350	39,226	51,677	41,734	59,194
Largest contig	25,401	474,180	172,683	50,052	688,925	126,557
Total length	36,029,448	37,341,447	76,326,026	66,984,094	70,838,814	94,470,748
N50	1,231	7,066	4,253	1,591	2,838	2,382

MEDIUM	Velvet	Ray	SPAdes	MEGAHIT	metaSPAdes	Trinity
# contigs	22,246	17,421	45,074	37,579	41,864	59,258
Largest contig	52,173	442,619	184,495	62,496	688,925	120,871
Total length	26,584,994	37,998,115	76,946,301	47,137,002	71,305,149	94,588,910
N50	1,318	7,254	2,893	1,492	2,865	2,388

HIGH	Velvet	Ray	SPAdes	MEGAHIT	metaSPAdes	Trinity
# contigs	22,686	17,364	42,987	51,749	41,505	59,108
Largest contig	56,951	303,232	172,683	66,255	542,864	120,871
Total length	27,444,611	37,964,392	77,214,295	67,927,289	72,257,839	96,105,718
N50	1,355	6,760	3,262	1,629	3,063	2,470

VERY HIGH	Velvet	Ray	SPAdes	MEGAHIT	metaSPAdes	Trinity
# contigs	32,332	17,048	41,502	51,459	41,392	58,988

**Largest contig**	56,951	467,217	172,683	91,584	464,577	168,064
**Total length**	37,378,587	41,056,751	76,752,084	67,809,374	72,108,940	96,703,227
**N50**	1,295	11,883	3,485	1,638	3,080	2,520

**Real data scenario**	**Velvet**	**Ray**	**SPAdes**	**MEGAHIT**	**metaSPAdes**	**Trinity**
**# contigs**	110,673	48,047	85,453	105,763	92,846	156,257
**Largest contig**	47,990	582,509	556,300	295,746	400,849	78,113
**Total length**	151,747,721	145,421,628	242,507,147	232,009,297	239,314,901	284,725,193
**N50**	1,752	15,515	12,135	5,044	8,114	3,046

Table S2. Mapping rate for the simulated scenario and the real dataset.

		**Simulated Scenario**			**Real data scenario**
**Assembly**	**”Low”**	**”Medium”**	**”High”**	**”Very high”**	**Illumina dataset**
**MEGAHIT**	73.97%	81.13%	67.80%	62.70%	86.83%
**metaSPAdes**	89.92%	90.06%	91.39%	92.34%	92.40%
**Trinity**	86.82%	88.23%	86.52%	83.57%	91.56%
**SPAdes**	89.83%	89.96%	89.93%	88.58%	92.38%
**Ray**	83.35%	83.93%	83.21%	89.71%	90.13%
**Velvet**	64.22%	69.37%	61.78%	49.06%	74.83%

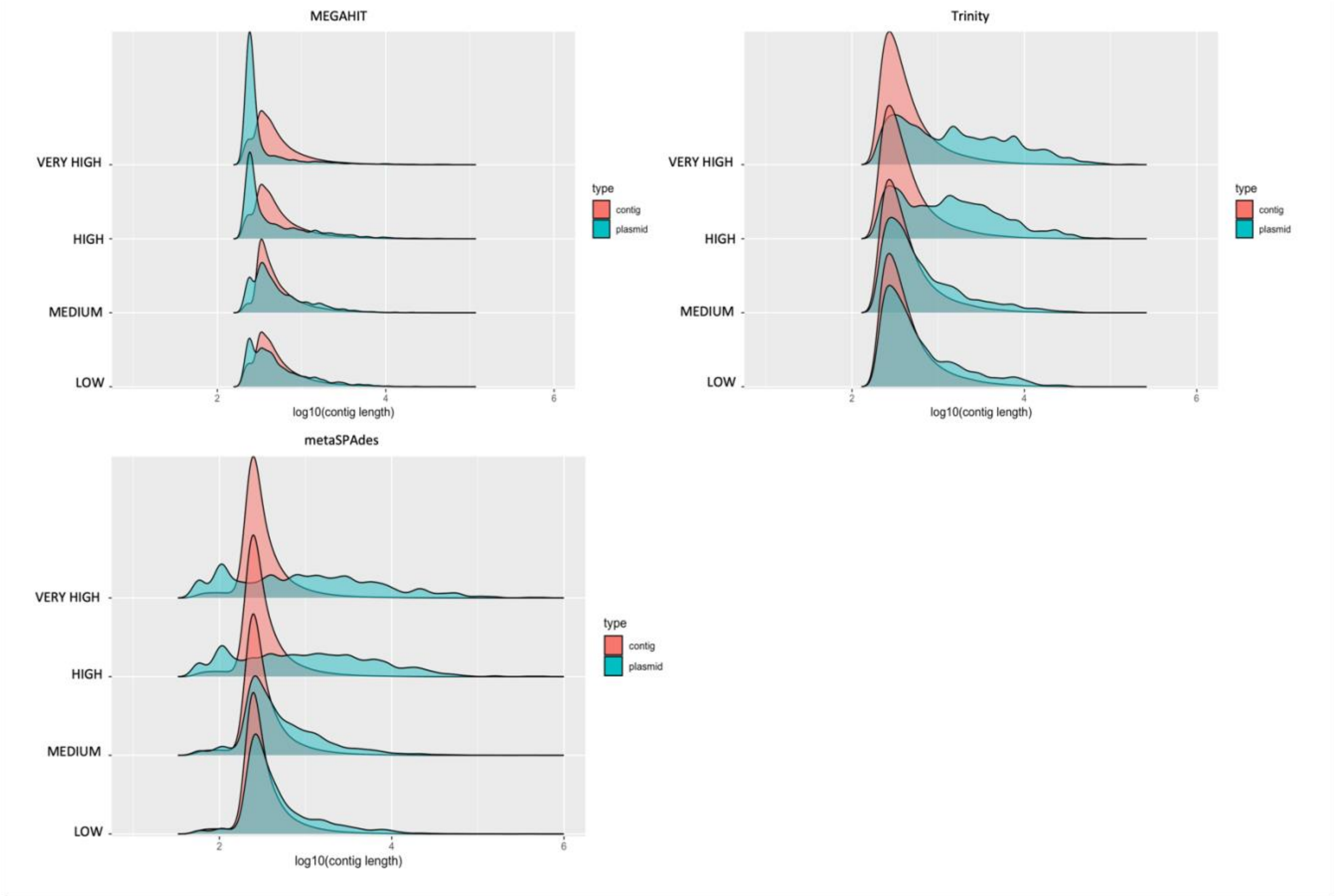

Figure S1. Comparison of length distribution of the contigs generated from the simulated reads in blue (corresponding to the plasmids) and the rest of the assembly in red. Contig length represented as log10.

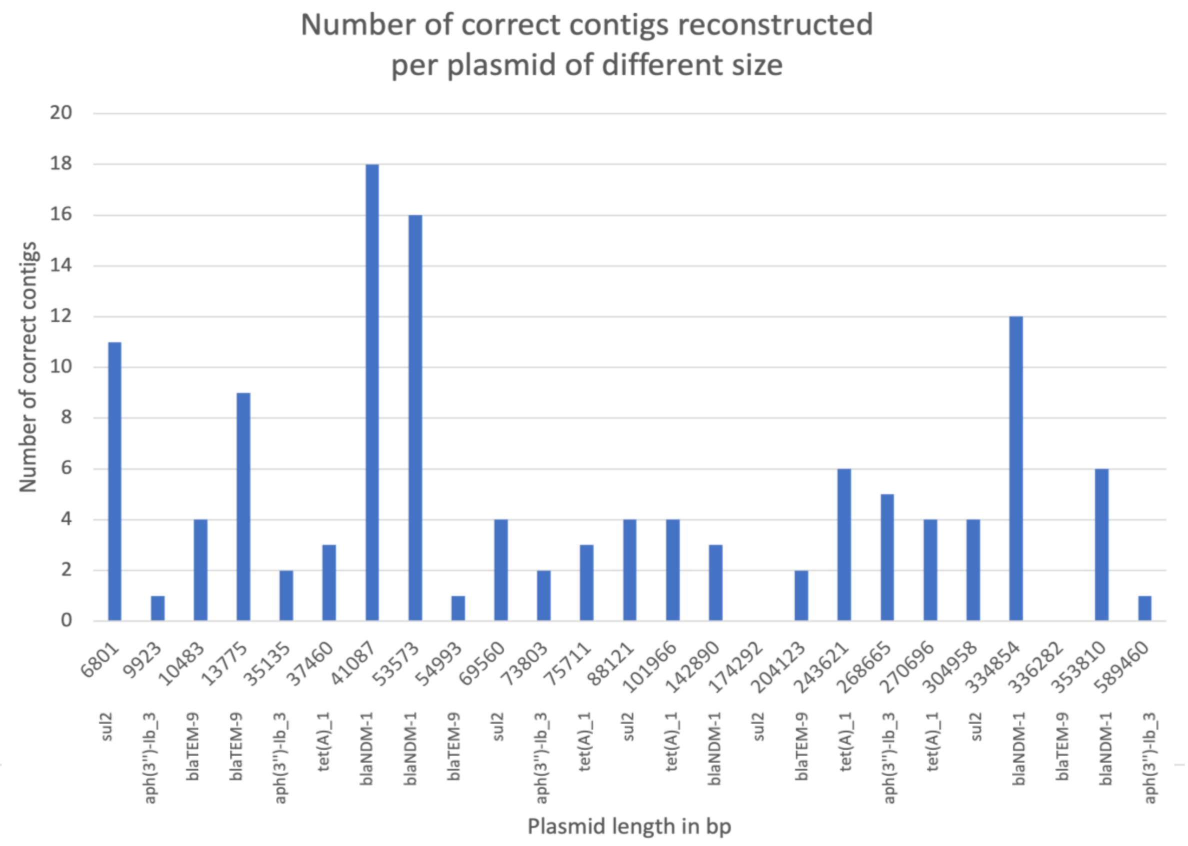

Figure S2. Number of correct contigs per plasmid of different size.

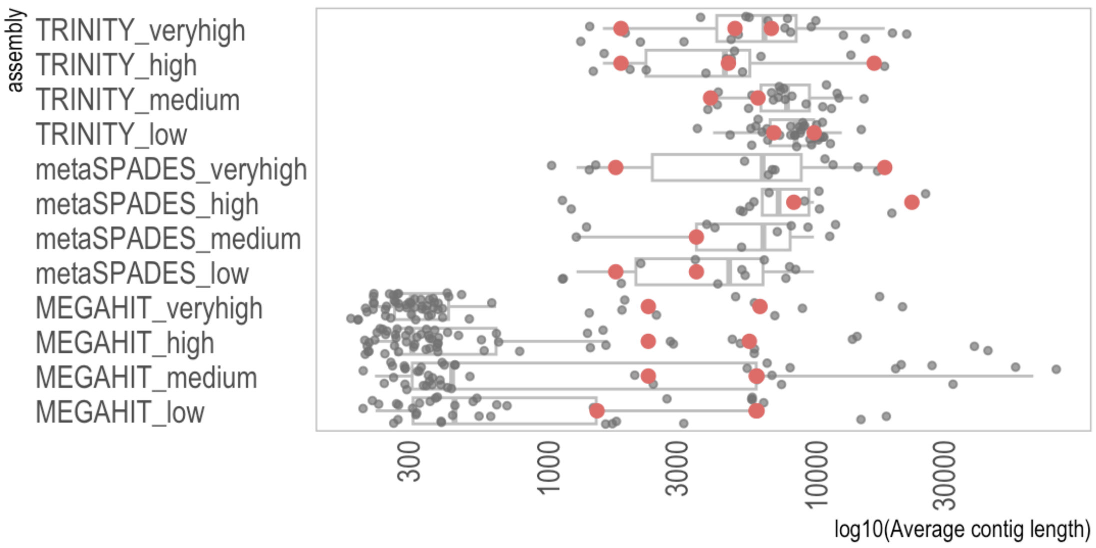

Figure S3. Length distribution of contigs with ARGs assembled from plasmid-derived reads only. Contigs with correct genomic context, only containing full ARGs, are marked with red dots.

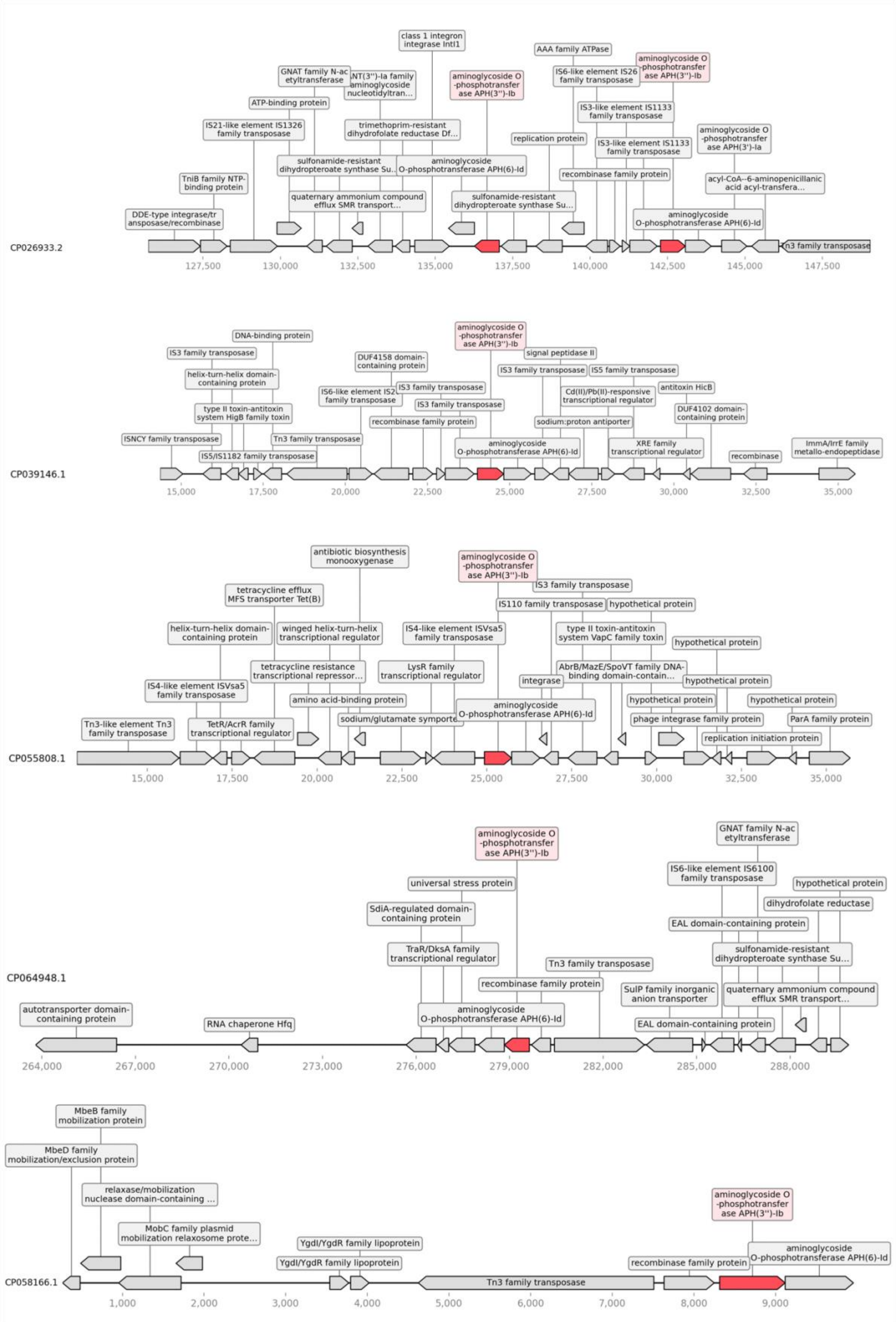

Figure S4 Genomic contexts around *aph(3'')-Ib* on five different plasmids. The figures depict 10 000 bp upstream and downstream of the ARG. The Figures were created using DNA Features Viewer (doi: 10.1093/bioinformatics/btaa213).

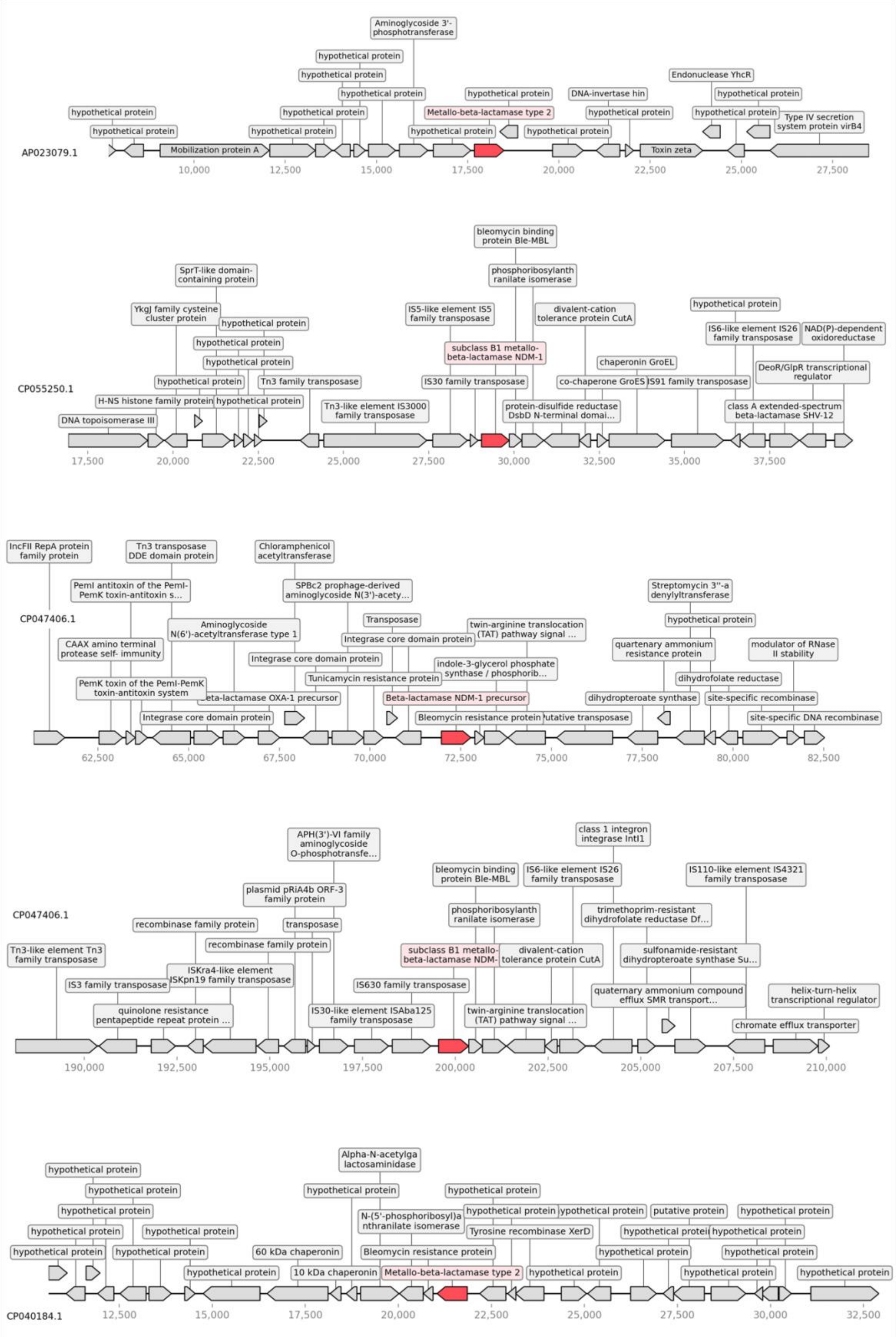

Figure S5 Genomic contexts around *blaNDM* on five different plasmids. The figures depict 10 000 bp upstream and downstream of the ARG. The Figures were created using DNA Features Viewer (doi: 10.1093/bioinformatics/btaa213).

Table S3. Correct genomic contexts assembled from Illumina short reads and compared to PacBio long reads as a reference.

Assembler	Number of unique contigs matching PacBio reads (98% identity, 100% coverage)	Number of different contexts the contigs match to	Average contig length, bp
Trinity	10	10	2737
SPAdes	5	36	1555
MEGAHIT	5	30	1190
metaSPAdes	5	34	1061
Velvet	1	3	953
TriMetAss	1	5	652
Ray	3	26	968
